# CHANGES IN D3CR MUSCLE MASS, WALKING SPEED, GRIP STRENGTH, AND CHAIR STANDS PERFORMANCE IN THE OLDEST OLD MEN

**DOI:** 10.1093/geroni/igad104.1612

**Published:** 2023-12-21

**Authors:** Peggy Cawthon, Li-Yung Lui, Megan Hetherington-Rauth, Kristine Ensrud, Jane Cauley, Adam Santanasto, William Evans, Eric Orwoll

**Affiliations:** California Pacific Medical Center, Research Institute, San Francisco, California, United States; California Pacific Medical Center, San Francisco, California, United States; California Pacific Medical Center, San Francisco, California, United States; University of Minnesota, Minneapolis, Minnesota, United States; University of Pittsburgh, Pittsburgh, Pennsylvania, United States; University of Pittsburgh, Pittsburgh, Pennsylvania, United States; University of California, Berkeley, Berkeley, California, United States; OHSU, Portland, Oregon, United States

## Abstract

One tenet in sarcopenia research is that strength is lost substantially more quickly than muscle mass with age. This assertion is mostly supported by data that uses indirect proxies for muscle mass, such as lean mass from dual energy x-ray absorptiometry (DXA). We posit that changes in direct estimates of muscle mass, such as muscle mass determined by d3-creatine dilution (D3Cr muscle mass), will be similar in magnitude to changes in strength and physical performance. In men who completed the Year 14 and Year 20 visits (N=220, Year 14 mean age: 82.9 yrs) of the MrOS study, we measured change in D3Cr muscle mass (kg), walking speed (m/s), grip strength (kg), and five repeat chair stand performance (stands/10 seconds). Over an average of 6.1 years of follow-up, men had extensive declines in D3Cr muscle mass (-3.29 kg, -12.8%), grip strength (-5.4 kg,-15.1%), walking speed (-0.23 m/s, -17.8%), and chair stands performance (-1.0 stands/10 seconds,-9.0%). Annualized percent change was of similar magnitude for D3Cr muscle mass (-2.2 %/year), grip strength (-2.5 %/year), walking speed (-2.9%/yr); with slightly lesser decline in chair stands performance (-1.5%/year). Change in D3Cr muscle mass was correlated with change in grip strength (r=0.22, p<.05) but not with change in walking speed or chair stand performance. Our finding that changes in muscle mass and strength were of similar magnitude and intercorrelated suggest that muscle mass itself has a more important role in loss of strength with age than has been previously considered, at least in the oldest-old men.

